# Achieving low intraocular pressure in normal-tension glaucoma with the Preserflo MicroShunt: comparison with primary open-angle glaucoma and the XEN-45 Gel Stent

**DOI:** 10.1186/s12886-026-05254-1

**Published:** 2026-08-21

**Authors:** Emil Nasyrov, Lasse Wolfram, Felix F. Reichel, Caroline J. Wenzel, Bogomil Voykov

**Affiliations:** Centre for Ophthalmology, University Hospital Tuebingen, Elfriede-Aulhorn-Str. 7, 72076 Tuebingen, Germany

**Keywords:** Normal-tension glaucoma, Preserflo MicroShunt, XEN Gel Stent, Primary open-angle glaucoma, Minimally invasive glaucoma surgery

## Abstract

**Background:**

Achieving low intraocular pressure (IOP) while minimizing surgical risk remains a central challenge in the management of normal-tension glaucoma (NTG). This study aimed to evaluate the efficacy and safety of the Preserflo MicroShunt (PMS) in NTG compared with primary open-angle glaucoma (POAG) and the XEN-45 Gel Stent.

**Methods:**

In this retrospective dual-comparison study, the primary outcomes were 2-year complete success (≥ 30% reduction in IOP from baseline and ≤ 12 mmHg without medication) and hazard ratios (HRs) for surgical failure. Kaplan–Meier analyses and univariate Cox models were used to compare NTG PMS and POAG PMS patients (1:1 matched cohort; PMS analysis) and exploratory multivariable models for consecutive NTG PMS and NTG XEN-45 patients (NTG analysis). Secondary outcomes included bleb revision and complication rates.

**Results:**

In the PMS analysis, 50 NTG patients were matched to 50 POAG patients. Two-year success rates were 69% and 45%, respectively (*p* = 0.0277). NTG diagnosis was associated with a lower risk of failure (HR 0.48, 95% CI 0.24–0.96) and fewer bleb revisions. In the NTG analysis, 45 PMS patients were compared with 23 XEN-45 patients. Two-year success rates were 71% and 17%, respectively (*p* < 0.0001). PMS implantation was associated with a lower risk of failure (HR 0.25, 95% CI 0.12–0.53) and fewer needling procedures. No sight-threatening complications occurred in any group.

**Conclusions:**

PMS implantation enabled a high rate of sustained low IOP control, with significantly higher success rates than in matched POAG patients and NTG XEN-45 patients, while requiring fewer postoperative interventions. These findings indicate that the PMS might achieve lower IOP thresholds in NTG than previously expected requiring evaluation in larger prospective studies.

**Supplementary Information:**

The online version contains supplementary material available at 10.1186/s12886-026-05254-1.

## Introduction

Reduction of intraocular pressure (IOP) is the only therapeutically modifiable factor in patients with normal-tension glaucoma (NTG) [1, 2]. However, achieving sufficiently low IOP while minimizing risks remains challenging with current surgical approaches. While trabeculectomy can achieve substantial IOP reduction, it is associated with a higher risk of potentially sight-threatening complications, particularly in NTG [3–6].

Minimally invasive bleb surgery (MIBS) procedures, including the Preserflo MicroShunt (PMS), have demonstrated efficacy comparable to trabeculectomy with a favourable safety profile in primary open-angle glaucoma (POAG) [7]. However, glaucoma specialists appear less likely to use the PMS in treating NTG [8, 9]. This may be related to the limited evidence regarding its efficacy in NTG, as well as the limited ability of another MIBS device, the XEN-45 Gel Stent, to achieve low postoperative IOP levels in these patients [10, 11]. In POAG, one-year postoperative IOP values of approximately 14–15 mmHg have been reported after PMS implantation, which may discourage its use in NTG, where substantially lower IOP levels are typically required [12]. To date, evidence for the use of PMS in NTG is scarce. Recently, one-year outcomes in 13 NTG patients reported an IOP reduction of 40% [13]. Therefore, the efficacy and safety of PMS in NTG remain insufficiently investigated, and its clinical role in this patient population has yet to be defined. This study aimed to determine whether PMS implantation can reliably achieve low IOP thresholds in NTG and to compare outcomes with matched POAG patients and NTG patients treated with the XEN-45 Gel Stent.

## Methods

This retrospective study consisted of two parts. First, a matched cohort study was conducted on patients with NTG and POAG in a 1:1 ratio who underwent PMS implantation. Second, an unmatched comparison was undertaken between consecutive NTG patients who received the PMS and consecutive NTG patients who received the XEN-45 Gel Stent.

NTG was defined as open-angle glaucoma with an IOP consistently ≤ 21 mmHg, either with or without topical medication, in accordance with the EGS Guidelines [14]. The highest recorded untreated IOP was obtained either from diurnal IOP measurements performed at our centre or from referring ophthalmologists’ records prior to initiation of IOP-lowering therapy and is reported in Tables 1 and 2. All patients underwent gonioscopy to confirm an open angle, and secondary causes of glaucoma were excluded. In case of atypical presentation, other aetiologies were ruled out by performing a neuro-ophthalmological assessment including cranial MRI. Patients with and without previous glaucoma or cataract surgery were eligible for inclusion.

**Table 1:** Demographic and clinical characteristics and surgical history of patients receiving the PMS

Characteristic	NTG *n* = 50 eyes of 50 patients	POAG control *n* = 50 eyes of 50 patients	*P*-value	SMD pre- match	SMD post-match
Median age (IQR), years	71 (64–77)	71.5 (63–77)	0.9986^a^	0.061	0.029
Female sex, %	70	62	0.5269^b^	0.522	0.169
White ethnicity, %	100	100			
Pseudophakia, %	50	48	>0.9999^b^	0.173	0.040
Laterality, % right eye	50	62	0.3138^2^		
Median refractive error (IQR), dpt spherical equivalent	−1.25 (−3.75 to 0)	−1 (−2.5 to 0)	0.6477^a^	0.043	0.081
Median BCVA (IQR), logMAR	0 (0−0.2)	0.1 (0−0.2)	0.6619^1^	0.346	0.048
Median Mean Deviation (IQR), dB	−8.6 (−15 to −4.2)	−10.5 (−15.8 to −5.4)	0.4467^a^	0.130	0.117
Median global ppRNFLT (IQR), µm	57 (46−65)	54 (47−68)	0.7186^a^		
Median preoperative medicated IOP (IQR), mmHg	19.5 (18–21)	20 (18–21)	0.6291^a^	1.567	0.047
Median number of preoperative medications (IQR)	3 (2–4)	3 (2–4)	0.5227^a^	0.271	0.132
Median postoperative follow-up time (IQR), months	21 (12–32)	22 (10–33)	0.4949^a^		
Median untreated max IOP (IQR), mmHg	20 (19–21)	29 (26–32)	**<0.0001** ^**a**^		
Median pre-operative duration of glaucoma care (IQR), months	67 (24–135)	26 (6–100)	0.0617^a^		
**Previous glaucoma procedures**,** %**			0.8758^c^	0.323	0.148
None	74	72			
Laser trabeculoplasty	6	10			
Angle-based procedures	4	6			
XEN-45 implantation	10	6			
Trabeculectomy	6	6			

Preoperative IOP and medications at the time of indication for surgery. The number of medications used was calculated based on the individual active agents. Duration of documented glaucoma care at our centre; total disease duration may be longer for patients referred from external providers. BCVA, best-corrected visual acuity; IQR, interquartile range; NTG, normal-tension glaucoma; PMS, Preserflo MicroShunt; POAG, primary open-angle glaucoma; ppRNFLT, peripapillary retinal nerve fibre layer thickness; SMD, standardised mean difference. Statistical tests: a = Mann–Whitney U test; b = Fisher’s exact test; c = chi-square test. P-values <0.05 are bolded

**Table 2:** Demographic and clinical characteristics and surgical history of patients with NTG

Characteristic	NTG PMS *n* = 45 eyes of 45 patients	NTG XEN-45 *n* = 23 eyes of 23 patients	*P*-value	SMD unmatched
Median age (IQR), years	71 (63–77)	68 (59–74)	0.1925^a^	0.247
Female sex, %	68.9	69.6	>0.9999^b^	0.006
White ethnicity, %	100	100		
Pseudophakia, %	48.9	30.4	0.1970^b^	0.259
Laterality, % right eye	48.9	34.8	0.3104^b^	
Median refractive error (IQR), dpt spherical equivalent	−1 (−3.25 to 0)	Not available		
Median BCVA (IQR), logMAR	0 (0−0.2)	0.1 (0−0.1)	0.9358^a^	0.083
Median Mean Deviation (IQR), -dB	-8.6 (-14.9 to -4.2)	-8.3 (-13.9 to -2.5)	0.3586^a^	0.203
Median global ppRNFLT (IQR), µm	57 (46−65)	54 (44−61)	0.2323^a^	
Median preoperative medicated IOP (IQR), mmHg	20 (18–21)	20 (18–21)	0.7141^a^	0.049
Median number of preoperative medications (IQR)	3 (2–4)	2 (1–4)	0.1780^a^	0.338
Median postoperative follow-up time (IQR), months	23 (12–33)	43 (33–54)	**<0.0001** ^**a**^	
Median untreated max IOP (IQR), mmHg	20 (19–21)	20 (18–20)	0.1535^a^	
Median pre-operative duration of glaucoma care (IQR), months	52 (16–134)	65 (35–153)	0.4187^a^	
**Previous glaucoma procedures**,** %**			0.6961^c^	0.179
None	82.2	73.9		
Laser trabeculoplasty	6.7	13		
Angle-based procedures	4.4	8.7		
Trabeculectomy	6.7	4.3		

Preoperative IOP and medications at the time of indication for surgery. The number of medications used was calculated based on the individual active agents. Duration of documented glaucoma care at our centre; total disease duration may be longer for patients referred from external providers. BCVA, best-corrected visual acuity; IQR, interquartile range; NTG, normal-tension glaucoma; PMS, Preserflo MicroShunt; POAG, primary open-angle glaucoma; ppRNFLT, peripapillary retinal nerve fibre layer thickness; SMD, standardised mean difference. Statistical tests: a = Mann–Whitney U test; b = Fisher’s exact test; c = chi-square test. P-values <0.05 are bolded

The exclusion criteria for both parts of the study were a follow-up period of less than 6 months and, for the second part, previous XEN-45 Gel Stent implantation in NTG patients receiving the PMS and vice versa. A flow diagram of patient selection and study design is provided in Supplementary Figure S1. The study was conducted in accordance with the tenets of the Declaration of Helsinki. The requirement for patient consent for the use of clinical data was waived by the ethics committee of the University of Tuebingen due to its retrospective design (project numbers 074/2023BO2 and 378/2021BO2).

### Surgical technique and perioperative management

Implantation of the PMS and XEN-45 Gel Stent was performed by a single experienced glaucoma surgeon (BV) under topical or general anaesthesia. Perioperative management was conducted as previously described [11, 15]. XEN-45 implantation was performed between July 2016 and March 2021; PMS implantation between April 2021 and March 2025. Mitomycin C (MMC) at a concentration of 0.2 mg/ml was injected as a 25 µl bubble before XEN implantation into the subconjunctival space and moved over the supranasal quadrant or applied in PMS implantation for 3 min using sponges under the conjunctiva following a fornix-based dissection in the supertemporal quadrant.

Preoperative IOP was assessed under maximally tolerated IOP-lowering medication at the time when the indication for surgery was made. IOP-lowering eyedrops were stopped 2 weeks prior to surgery. Preservative-free dexamethasone eyedrops were applied five times daily starting two weeks preoperatively. Postoperatively, the frequency was increased to hourly and tapered over three months. Follow-ups were conducted on postoperative day 1, 2, and week 2; at months 3, 6, 9, and 12; and then every six months. These visits involved a full ophthalmological examination, including changes in best-corrected visual acuity (BCVA) from baseline, IOP measurement using Goldmann applanation tonometry, slit-lamp examination and seidel testing for bleb leakage. The presence of choroidal effusions and hypotony maculopathy was assessed by fundus examination and, where indicated for the latter, by optical coherence tomography.

Conjunctival bleb needling was performed in case of IOP elevation above the target range, associated with bleb encapsulation or Tenon cyst formation in the absence of other identifiable causes of IOP rise. Incisional bleb revision was indicated either when needling failed to restore adequate filtration or was performed primarily in cases of a dense subconjunctival fibrosis, suggested by the PMS not being visible under the conjunctiva. The indication was made by the treating surgeon and did not change throughout the study period.

### Study outcome measures and baseline characteristics

The primary outcome measures were surgical success at 2 years and the hazard ratio (HR) for failure. Success was defined as a ≥ 30% reduction in IOP from baseline with an IOP of ≤ 12 mmHg, in accordance with the guidelines of the World Glaucoma Association [16].

No lower IOP limit (e.g. ≥ 6 mmHg) was applied in the definition of success. Instead, hypotony was defined clinically by the presence of choroidal effusion or hypotony maculopathy, and the absence of these findings was required for success [17]. The incidence of numerical hypotony (< 6 mmHg) was recorded and is reported as secondary outcome. If patients did not meet the IOP and hypotony success criteria at two consecutive visits starting from month 3, failure was recorded at the first visit at which the criteria were not met. Furthermore, the need for further glaucoma surgery, including incisional bleb revisions, loss of light perception and the need for oral acetazolamide was considered a failure. Needling procedures were not considered failures [16, 18]. Additional success categories were considered for a ≥ 25% reduction in IOP from baseline with an IOP of ≤ 15 mmHg and a ≥ 40% reduction in IOP from baseline with an IOP of ≤ 9 mmHg. Complete success was defined as the achievement of the success criteria without IOP-lowering medication. Qualified success was defined as meeting the success criteria regardless of the use of topical IOP-lowering medication, provided that the same or a lower number of agents was used compared with baseline.

The secondary outcome measures included median IOP, change in IOP from baseline, the proportion of medication-free eyes, intervention rates (including needlings, incisional bleb revisions, and further glaucoma surgeries), and complication rates.

Patients’ characteristics and histories, IOP readings and other measurements, the presence of choroidal effusion and details of hypotony interventions, needlings, and incisional bleb revisions were retrieved from the electronic medical record system. Autorefraction was performed using the ARK-1s autorefractor (NIDEK, Gamagori, Japan) to assess refractive error. The spherical equivalent refractive error of phakic patients was retrieved from visits at which no cataract was documented. Conversely, the refractive error of pseudophakic patients was obtained from visits prior to cataract surgery.

### Matching

Eligible NTG patients who received the PMS were matched to POAG patients in a 1:1 ratio using propensity score matching. Propensity scores were estimated using a logistic regression model with NTG diagnosis as the dependent variable and the following baseline covariates as independent variables: age, sex, preoperative IOP, number of preoperative medications, mean deviation (MD), spherical equivalent, pseudophakia, and previous glaucoma surgical procedures. Nearest-neighbour matching without replacement and without a caliper restriction was applied. Multiple matching strategies were evaluated, including 1:1, 1:2, and 1:3 ratios, with and without a caliper of 0.2 on the logit of the propensity score. The 1:1 approach without caliper was selected because it retained all NTG patients while achieving adequate covariate balance. Higher matching ratios resulted in substantially worse balance for preoperative IOP, and caliper-based matching excluded a relevant proportion of NTG patients without meaningful improvement in overall balance. Balance between groups was assessed using standardised mean differences (SMDs). An SMD below 0.1 was considered to indicate excellent balance, and an SMD below 0.2 was considered to indicate adequate balance [19, 20]. SMDs before and after matching are reported in Table 1. Matching was performed using the MatchIt package in R version 4.4.0 (R Foundation for Statistical Computing, Vienna, Austria).

### Statistical analysis

Descriptive statistics were used to summarise the demographic and clinical characteristics. Continuous variables were assessed for normality using the Shapiro–Wilk test and were found to be non-normally distributed. These variables were reported as medians and interquartile ranges (IQRs). Categorical variables were summarised as percentages. Within-group comparisons for continuous variables were performed using the Wilcoxon matched-pairs signed-rank test. Between-group comparisons were conducted using the Mann–Whitney U test. Categorical variables were compared using the chi-square test or Fisher’s exact test, as appropriate.

Kaplan–Meier survival analyses were performed in the matched sample and compared using the log-rank test. To estimate the effect of diagnosis on surgical failure, a univariate Cox proportional hazards model was fitted with diagnosis (NTG vs. POAG) as the sole independent variable. To account for the matched study design, robust sandwich variance estimation was applied with clustering on the matched pair identifier, as recommended for propensity score–matched survival analyses [19]. Hazard ratios (HRs) with corresponding 95% confidence intervals (CIs) and two-sided p-values were reported. Given the adequate covariate balance achieved after matching (all SMDs < 0.2), additional multivariable adjustment was not performed [19]. Three sensitivity analyses were conducted for the PMS analysis. In the first, incisional bleb revisions were not considered failures. In the second, an alternative success definition based solely on percentage IOP reduction from baseline (≥ 40%, ≥ 30%, and ≥ 25%) was applied. In the third, both modifications were combined.

For the comparison of NTG patients receiving the PMS versus the XEN-45 Gel Stent, Kaplan–Meier survival analyses were performed and compared using the log-rank test. Because this comparison was unmatched, multivariable Cox proportional hazards models were fitted to adjust for baseline differences. Given the limited number of failure events, the number of covariates was restricted to maintain an adequate events-per-variable ratio [21]. Models included the type of device (PMS vs. XEN-45) as the primary exposure variable and were adjusted for the number of preoperative medications and pseudophakia, which were selected based on the largest baseline imbalances between groups (SMDs > 0.2; Table 2). The number of failure events for each success category is reported in the results. For sensitivity analysis, administrative right truncation at 24 months was applied to account for the longer follow-up in the NTG XEN-45 group. For both analyses, no violation of the proportional hazards assumption was detected (Schoenfeld residuals, *p* > 0.05).

Time-to-event analyses for postoperative interventions were performed using Kaplan–Meier methods. Time to first needling, time to first incisional bleb revision, and time to first bleb revision of any type were analysed separately. Groups were compared using the log-rank test. The number of revisions was calculated per patient-year of follow-up.

All analyses were conducted using R version 4.4.0 (R Foundation for Statistical Computing) with the survival and survminer packages and GraphPad Prism version 10.2.0 (GraphPad Software, Boston, USA). A two-sided p-value *p* < 0.05 was considered statistically significant.

## Results

### Study patients

Table 1 summarises the demographic and clinical characteristics of the NTG and POAG groups in the PMS analysis. One NTG patient was excluded due to a follow-up of only two weeks, resulting in 50 NTG patients being included (Figure S1). These were matched to 50 POAG patients selected from 343 eligible controls. Baseline characteristics were comparable between matched groups (Table 1).

Table 2 presents the characteristics of the NTG PMS and NTG XEN-45 groups (NTG analysis). Five patients with the PMS had previously received a XEN-45 implant and were excluded, resulting in 45 NTG PMS patients and all 23 NTG XEN-45 patients, who were eligible, being included in the analysis (Figure S1). Baseline imbalances were most pronounced regarding preoperative medications, pseudophakia and age. The median (IQR) follow-up duration was significantly longer in the XEN-45 group (Table 2).

### Postoperative IOP changes after PMS implantation

**Fig. 1:**
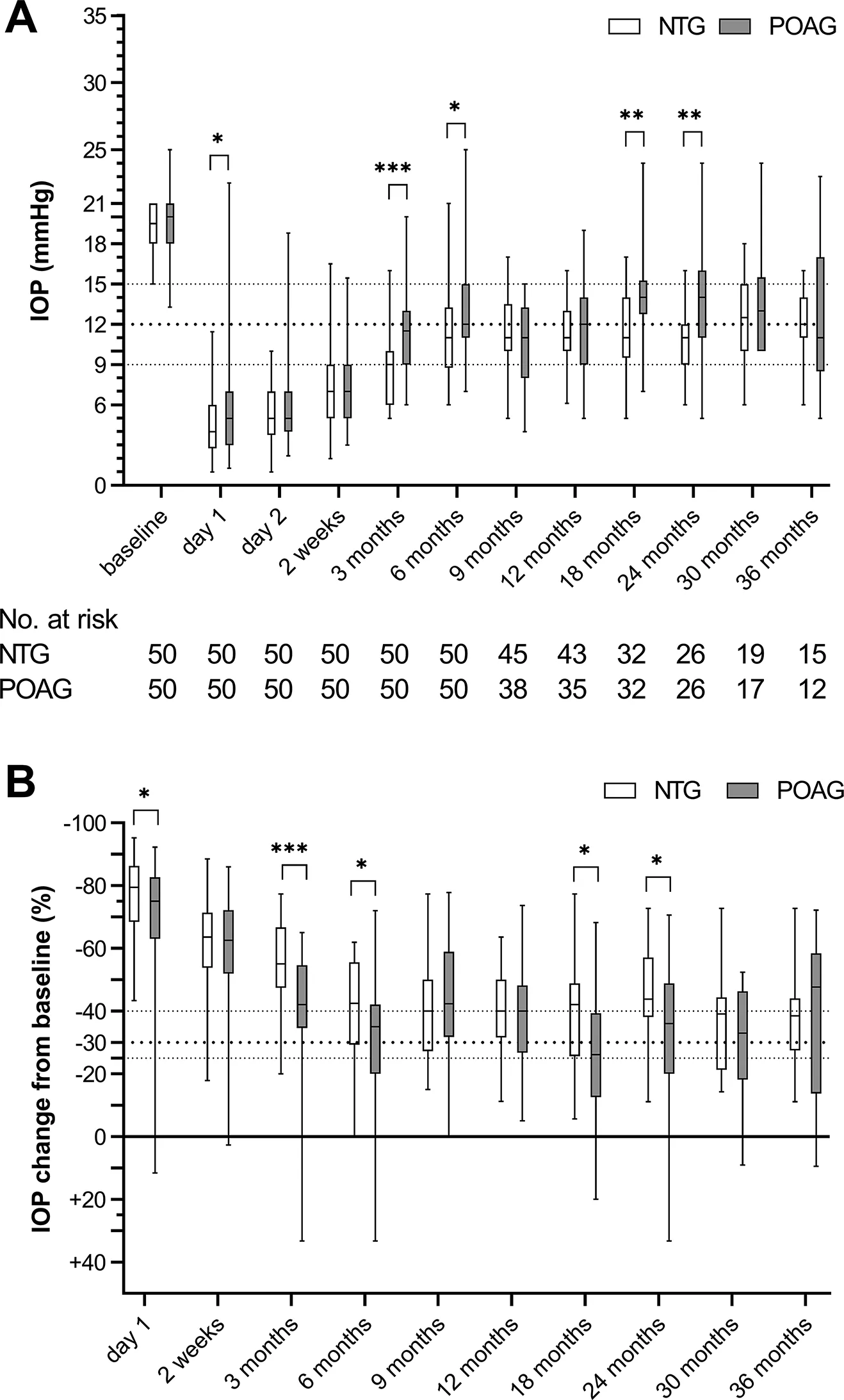
Postoperative IOP and IOP reduction after PMS implantation in patients with NTG and POAG. Postoperative median (IQR) IOP (**A**) and median IOP reduction from baseline (**B**) are plotted across postoperative visits. The POAG group is represented by grey bars and the NTG group by white bars. Error bars indicate the 95% confidence intervals. Horizontal dotted lines indicate relevant cut-off values regarding the success criteria. ^*^p < 0.05; ^**^p < 0.01; ^***^p < 0.001

PMS implantation resulted in a sustained reduction in IOP at all postoperative time points up to three years in both NTG and POAG groups (each *p* < 0.001; Wilcoxon matched-pairs signed-rank test; Fig. 1A). Median (IQR) IOP at 1, 2, and 3 years was 11 (10–13, *n* = 43), 11 (9–12, *n* = 26), and 12 (11–14, *n* = 15) mmHg in the NTG group and 12 (9–14, *n* = 35), 14 (11–16, *n* = 26), and 11 (9–17, *n* = 12) mmHg in the POAG group. Three-year results are reported for completeness but should be interpreted with caution given the reduced sample size. Median IOP was significantly lower and median IOP reduction higher in the NTG group at day 1 and months 3, 6, 18 and 24 (Mann–Whitney U test; Fig. 1A and B). Median IOP reduction exceeded 40% at all visits up to 2 years in the NTG group (Fig. 1B). Median (IQR) IOP reduction from baseline in the NTG group was 40% (32–50), 44% (38–57), and 39% (27–44) after 1, 2, and 3 years. Corresponding reductions in the POAG group were 40% (27–48), 36% (20–49), and 48% (14–59).

The median (IQR) number of medications decreased to 0 (0–0) at 1, 2, and 3 years in both groups (each *p* < 0.001 vs. baseline; Wilcoxon matched-pairs signed-rank test). In the NTG group, 91%, 93%, and 87% of eyes were medication-free at years 1, 2, and 3, respectively, compared with 97%, 88%, and 100% in the POAG group (all *p* > 0.05; Fisher’s exact test).

### Surgical success of the PMS in patients with NTG versus POAG

**Fig. 2:**
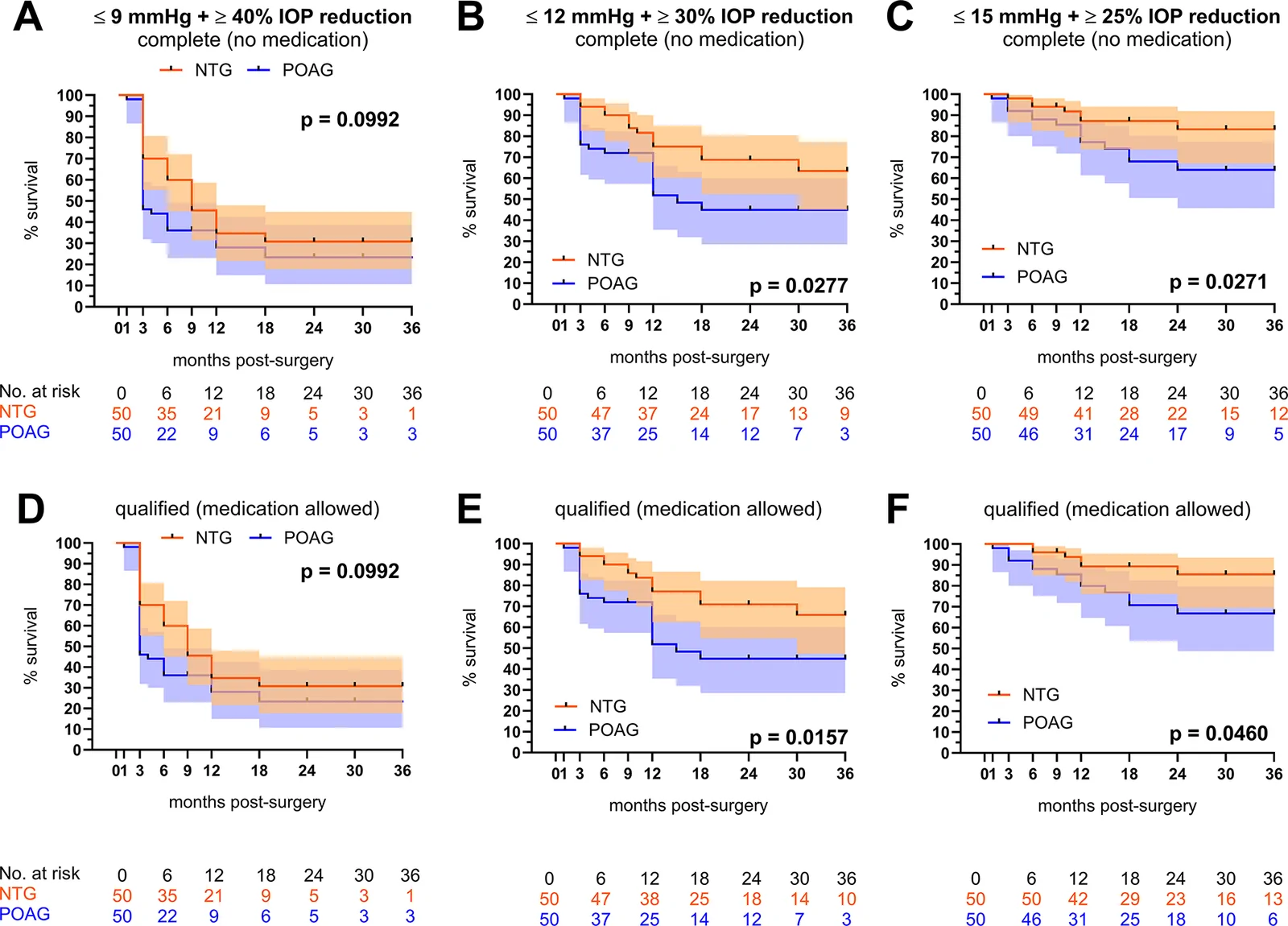
Kaplan–Meier survival curves after PMS implantation in patients with NTG versus POAG. Kaplan–Meier estimates were used to compare complete and qualified surgical success for category 1 (**A** and **D**), category 2 (**B** and **E**), and category 3 (**C** and **F**) between patients with NTG (orange) and POAG (blue) using the log-rank test. The 95% confidence intervals are represented in lighter orange and blue colours, respectively

Figure 2 shows the Kaplan–Meier survival curves for complete and qualified success categories. Complete success for category 1 (IOP ≤ 9 mmHg with ≥ 40% reduction without medication) was indicated by a trend to be more frequent in the NTG group, achieved by 35% (95% CI 22–48) at year 1 and 31% (18–45) at years 2 and 3 (*p* = 0.0992; log-rank test; Fig. 2A). In the POAG group, rates were 28% (15–43) at year 1 and 23% (15–43) at years 2 and 3.

Complete success for the main outcome, category 2 (IOP ≤ 12 mmHg with ≥ 30% reduction) was significantly higher in the NTG group, reaching 75% (60–85), 69% (53–81), and 64% (45–77) after 1, 2, and 3 years, respectively (*p* = 0.0277; Fig. 2B). In the POAG group, rates were 52% (35–66) after 1 and 45% (29–60) after 2 and 3 years.

For category 3 (IOP ≤ 15 mmHg with ≥ 25% reduction), complete success was achieved by 87% (74–94) at year 1 and 83% (67–92) at years 2 and 3 in the NTG group (*p* = 0.0271; Fig. 2C). Corresponding rates in the POAG group were 77% (61–87) at year 1 and 64% (46–77) at years 2 and 3.

Qualified success rates were identical to complete rates for category 1 and only slightly higher for categories 2 and 3, reflecting the high proportion of medication-free eyes (Fig. 2D–F). Qualified success was significantly higher in the NTG than POAG group across categories 2 and 3 (Fig. 2E–F). Table 3 shows the univariate Cox models for failure in each category. NTG diagnosis was associated with lower risk to fail success categories 2 and 3.

**Table 3:** Stratified univariate Cox regression for surgical failure following PMS implantation in NTG versus POAG

Success category	Events (*n*/*N*)	HR (95% CI)	*P*-Value (Cox)
≤9 mmHg, ≥40% (complete)	68/100	0.65 (0.39–1.09)	0.1026
≤9 mmHg, ≥40% (qualified)	68/100	0.65 (0.39–1.09)	0.1026
≤12 mmHg, ≥30% (complete)	38/100	**0.48 (0.24–0.96)**	**0.0391**
≤12 mmHg, ≥30% (qualified)	37/100	**0.44 (0.22–0.89)**	**0.0223**
≤15 mmHg, ≥25% (complete)	22/100	**0.38 (0.16–0.9)**	**0.0283**
≤15 mmHg, ≥25% (qualified)	19/100	**0.39 (0.16–0.93)**	**0.0340**

HR, hazard ratio; CI, confidence interval; NTG, normal-tension glaucoma; POAG, primary open-angle glaucoma; PMS, Preserflo MicroShunt. HR <1 indicates lower risk of failure for NTG relative to POAG. Robust sandwich variance estimation was applied with clustering on the matched pair identifier. Statistically significant results (*p* < 0.05) are shown in bold

### Complications and bleb revisions after PMS implantation

Table 4 summarises complication rates in NTG and POAG patients following PMS implantation which were similar between groups. Transient numerical hypotony (< 6 mmHg) occurred in 74% and 72% of patients in the early postoperative period. Beyond 3 months it was found on single, but not consecutive visits in only 6% of eyes in each group. Choroidal effusions required treatment in 12% of NTG and 10% of POAG patients with viscoelastic injections. Most received one injection, 2 NTG patients required 2 and 1 POAG patient 3, and all effusions resolved without permanent sequelae.

**Table 4:** Complication rates following PMS implantation in NTG versus POAG

Rate %	NTG *n* = 50 eyes of 50 patients	POAG control *n* = 50 eyes of 50 patients	*P*-value
Numerical hypotony (<6 mmHg) at any visit	74	72	>0.9999^a^
Numerical hypotony (<6 mmHg) at any visit >3 months	6	6	>0.9999^a^
Numerical hypotony (<6 mmHg) at 2 consecutive visits >3 months	0	0	>0.9999^a^
Hypotony maculopathy	0	0	>0.9999^a^
Choroidal effusion at any visit	16	16	>0.9999^a^
AC formation with viscoelastic	12	10	0.5733^a^
Intraluminal stenting	0	0	>0.9999^a^
Median days to resolution of choroidal effusion	10	7	0.7621^b^
Loss of ≥ 2 lines BCVA logMAR at last-follow-up	12	14	>0.9999^a^
AC haemorrhage	32	17	0.0585^1^
Bleb leakage	0	0	>0.9999^a^

AC, anterior chamber; BCVA; best-corrected visual acuity; NTG, normal-tension glaucoma; PMS, Preserflo MicroShunt; POAG, primary open-angle glaucoma. Statistical tests: a = Fisher’s exact test; b = Log-rank test. P-values <0.05 are bolded

The overall rate of bleb revisions (needling or incisional) was indicated by a trend to be higher in the POAG group (*p* = 0.0714; log-rank test). These were required in 7% and 15% of NTG patients and 19% and 38% of POAG patients at 1 and 2 years, respectively. The number of bleb revisions per patient-year was almost three times higher in the POAG group (0.108 vs. 0.310). Bleb needling rates were similar between NTG and POAG patients (*p* = 0.5751), required in 7% and 15% of NTG cases and 10% and 25% of POAG cases at 1 and 2 years, respectively. Incisional bleb revisions were significantly less frequent in the NTG group (2% vs. 9% at 1 year, 2% vs. 19% at 2 years; *p* = 0.0224). The numbers of needlings and incisional revisions per patient year was 0.089 and 0.02 in the NTG group, and 0.145 and 0.166 in the POAG group, respectively. No sight-threatening complications, such as hypotony maculopathy, suprachoroidal haemorrhage, or blebitis, were observed in either group. Two POAG patients required PMS exchange due to the PMS not demonstrating patency during incisional revision.

### Sensitivity analyses of the PMS comparison

Three prespecified sensitivity analyses were performed to assess the robustness of the findings (Figures S2-S4 and Tables S1-S3). In the first, incisional revisions were not considered failures (Fig. S2 and Table S1). NTG diagnosis remained significantly associated with lower failure risk for categories 2 and 3 (e.g., HR 0.48, 95% CI 0.23–0.99, and HR 0.29, 95% CI 0.10–0.82, for complete success, respectively), with effect estimates of similar or greater magnitude compared with the main analysis. In the second, absolute IOP thresholds were removed and success was defined by percentage IOP reduction alone (Fig. S3 and Table S2). Qualified success for category 1 (IOP and ≥ 40% reduction) was significantly higher in the NTG group. NTG diagnosis remained significantly associated with lower failure risk for ≥ 30% and ≥ 25% IOP reduction categories (HR 0.45, 95% CI 0.22–0.91, and HR 0.32, 95% CI 0.13–0.76, for complete success, respectively). In the third sensitivity analysis combining both modifications, results remained significant for categories 2 and 3 across complete and qualified success definitions and qualified success in category 1 remained significantly higher in the NTG group (Fig. S4 and Table S3). The direction and magnitude of the effect estimates were consistent across all sensitivity analyses.

### Postoperative IOP changes in patients with NTG following PMS implantation compared With XEN-45 implantation

**Fig. 3:**
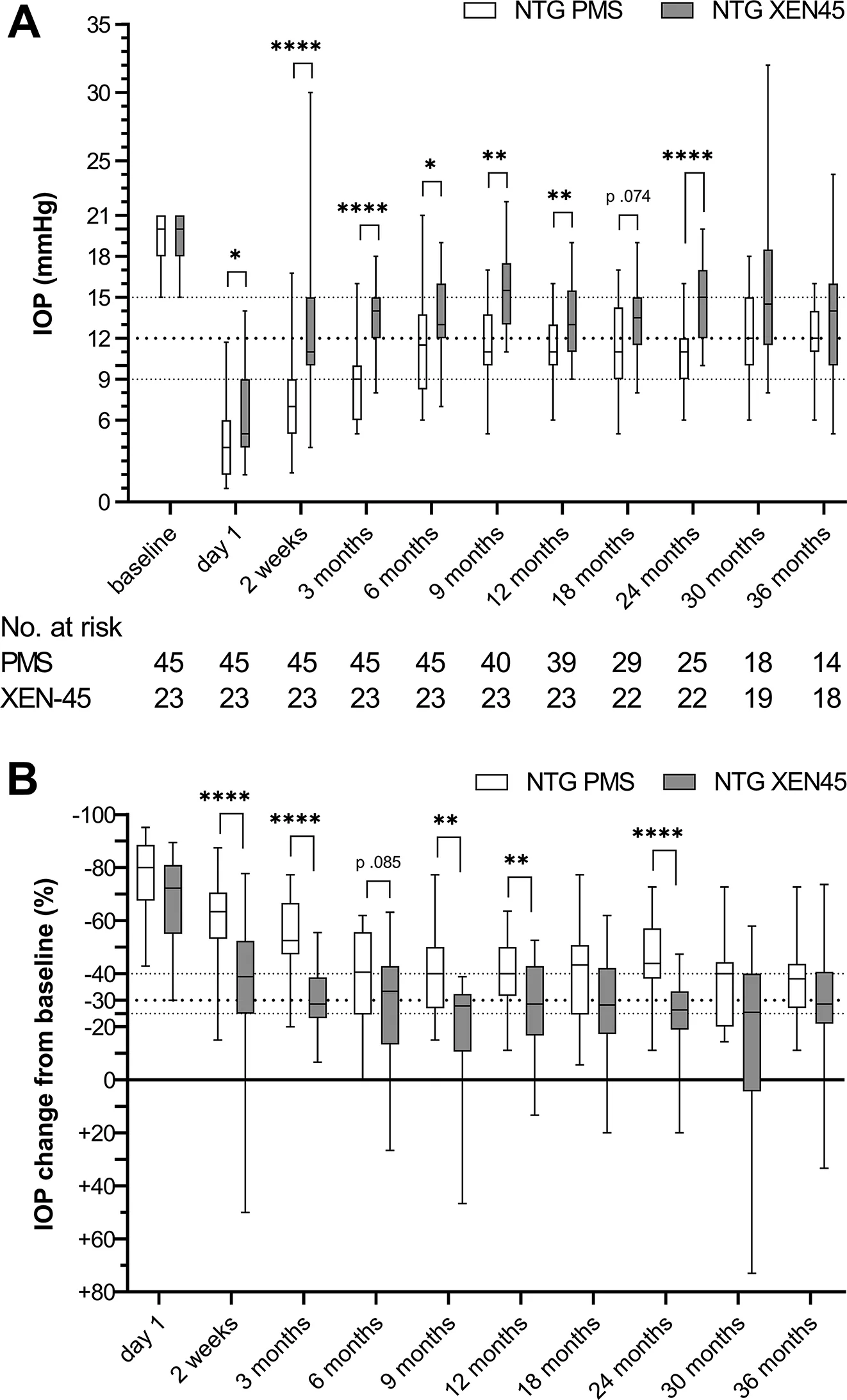
Postoperative IOP and IOP reduction after PMS implantation Versus XEN-45 implantation in patients with NTG. Postoperative median (IQR) IOP (**A**) and IOP reduction from baseline (**B**) are plotted across postoperative visits. The XEN-45 group is represented by grey bars and the PMS group by white bars. Error bars indicate the 95% confidence intervals. Horizontal dotted lines indicate relevant cut-off values regarding the success criteria. ^*^p < 0.05; ^**^p < 0.01; ^****^p < 0.0001

In the second part of the study, postoperative median IOP was significantly lower in the NTG PMS group than in the NTG XEN-45 group at most visits up to two years, except for month 18 where only a trend was observed (Mann–Whitney U test; Fig. 3A). Accordingly, IOP reduction from a comparable baseline IOP was greater in the NTG PMS group at most visits within the two-year follow-up period (Fig. 3B). Median IOP reduction exceeded 30% at all visits in the NTG PMS group but was frequently below 30% in the NTG XEN-45 group. Median (IQR) IOP reduction from baseline in the NTG PMS group was 40% (32–50), 44% (38–57), and 38% (27–44) after 1, 2, and 3 years, respectively. Corresponding reductions in the NTG XEN-45 group were 29% (17–43), 26% (19–33), and 29% (21–41).

Median (IQR) medication use decreased to 0 (0–0) at 1, 2, and 3 years in the NTG PMS group (each *p* < 0.001 vs. baseline; Wilcoxon matched-pairs signed-rank test). In the NTG XEN-45 group, medication use was 0 (0–0) at year 1 and 0 (0–1) at years 2 and 3 (each *p* < 0.01 vs. baseline). Medication-free rates at years 1, 2, and 3 were 90%, 92%, and 86% in the NTG PMS group and 83%, 68%, and 56% in the NTG XEN-45 group (all *p* > 0.05; Fisher’s exact test).

### Surgical success in patients with NTG following PMS implantation compared with XEN-45 implantation

**Fig. 4:**
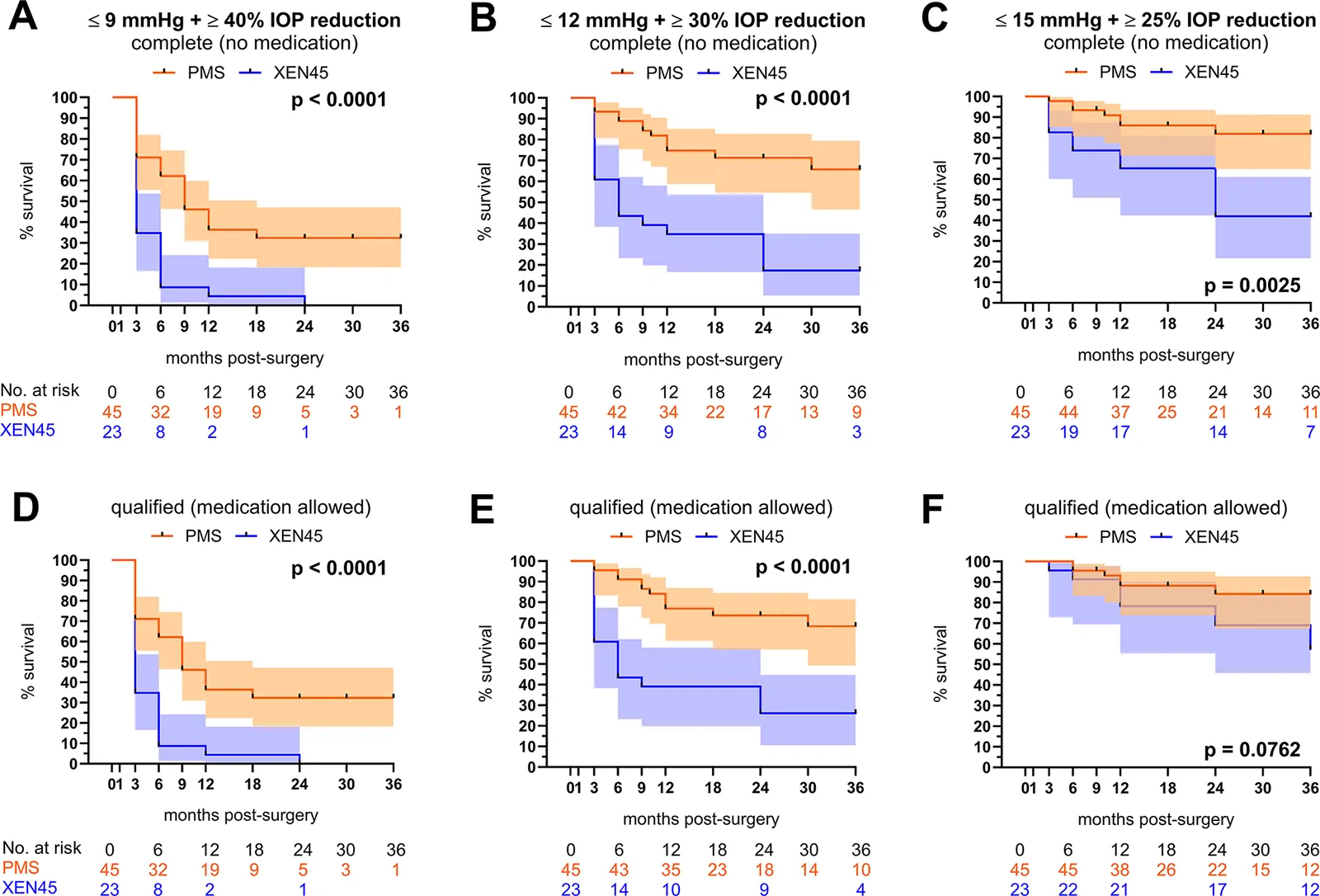
Kaplan–Meier survival curves after PMS implantation Versus XEN-45 implantation in patients with NTG. Kaplan–Meier estimates were used to compare complete and qualified surgical success for category 1 (**A** and **D**), category 2 (**B** and **E**), and category 3 (**C** and **F**) between NTG eyes that received the PMS (orange) and XEN-45 implant (blue) using the log-rank test. The 95% confidence intervals are represented in lighter orange and blue colours, respectively

Kaplan–Meier survival curves for complete and qualified success are shown in Fig. 4. Complete success for category 1 was achieved in 36% (95% CI 23–50) of NTG PMS patients at year 1 and 32% (18–47) at years 2 and 3. In contrast, rates in the NTG XEN-45 group were 4% (0.3–18) at year 1 and 0% at years 2 and 3 (*p* < 0.0001; log-rank test; Fig. 4A).

For category 2, complete success in the NTG PMS group was 75% (59–85), 71% (55–83), and 66% (47–80) after 1, 2, and 3 years. In the NTG XEN-45 group, rates were significantly lower at 35% (17–54) after 1 year and 17% (5–35) after 2 and 3 years (*p* < 0.0001; Fig. 4B).

For category 3, complete success rates were 86% (71–94) at year 1 and 82% (65–91) at years 2 and 3 in the NTG PMS group, compared with 65% (42–81) and 42% (22–61) in the NTG XEN-45 group (*p* = 0.0025; Fig. 4C).

Qualified and complete success rates were identical for category 1. For category 2, qualified success at 2 years was achieved in 74% (57–85) of PMS eyes compared with 26% (11–45) of XEN-45 eyes (*p* < 0.0001). For Category 3, qualified success was similar between groups (84% [67–93] vs. 69% [46–84] at year 2; *p* = 0.0762; Fig. 4E).

Exploratory multivariable Cox regression models, controlled for preoperative number of medications and pseudophakia, showed PMS implantation (vs. XEN-45) to be significantly associated with lower failure risk across all complete success categories and qualified success categories 1 and 2 (Table 5).

**Table 5:** Multivariable Cox regression for surgical failure following PMS versus XEN-45 implantation in NTG

Variable	≤9 mmHg, ≥40% (complete)	≤9 mmHg, ≥40% (qualified)	≤12 mmHg, ≥30% (complete)	≤12 mmHg, ≥30% (qualified)	≤15 mmHg, ≥25% (complete)	≤15 mmHg, ≥25% (qualified)
No. of events (n/N)	52/68	52/68	32/68	29/68	20/68	15/68
PMS (reference: XEN-45)	**0.28 (0.15–0.51); <0.0001**	**0.28 (0.15–0.51); <0.0001**	**0.25 (0.12–0.53); <0.0001**	**0.27 (0.12–0.58); 0.001**	**0.26 (0.1–0.67); 0.006**	0.38 (0.13–1.13); 0.082
Number of preoperative medications	1.02 (0.81–1.29); 0.852	1.02 (0.81–1.29); 0.852	0.87 (0.66–1.15); 0.339	0.88 (0.66–1.17); 0.383	0.99 (0.7–1.4); 0.957	1.17 (0.75–1.8); 0.491
Pseudophakia	1.27 (0.72–2.24); 0.411	1.27 (0.72–2.24); 0.411	0.85 (0.41–1.73); 0.648	0.61 (0.28–1.33); 0.213	1.05 (0.42–2.64); 0.922	0.81 (0.26–2.51); 0.717

Hazard ratios with (95% confidence interval); and p-values are shown for every variable in each model. NTG, normal-tension glaucoma; PMS, Preserflo MicroShunt. HR <1 indicates lower risk of failure for PMS relative to XEN-45. The model was adjusted for the number of preoperative medications and pseudophakia, which were selected based on baseline imbalances between groups. Statistically significant results (*p* < 0.05) are shown in bold

### Sensitivity analyses of the NTG comparison

To account for differences in follow-up duration between the NTG groups, a sensitivity analysis was performed with administrative right truncation at 24 months (median follow-up in the NTG PMS group 23 months). Kaplan–Meier survival curves up to 24 months were identical to those of the main analysis (Figure S5). Cox regression models yielded similar hazard ratios, with the type of device (PMS vs. XEN-45) remaining the only variable significantly associated with failure in our cohort (Table S4).

### Complications and bleb revisions in NTG patients following PMS compared with XEN-45 implantation

Complication rates are summarised in Table 6 which were similar between groups.

**Table 6:** Complication rates of patients with NTG following PMS Versus XEN-45 implantation

Rate % (eyes)	NTG PMS *n* = 45 eyes of 45 patients	NTG XEN-45 *n* = 23 eyes of 23 patients	*P*-value
Numerical hypotony (<6 mmHg) at any visit	72	56	0.1949
Numerical hypotony (<6 mmHg) at any visit >3 months	2.2	4.4	>0.9999
Numerical hypotony (<6 mmHg) at 2 consecutive visits >3 months	0	0	>0.9999
Hypotony maculopathy	0	0	>0.9999
Choroidal effusion at any visit	17.8	4.4	0.1530
AC formation with viscoelastic	13.33	4.4	0.4090
Loss of ≥ 2 lines BCVA logMAR at last-follow-up	11.1	21.7	0.2880^a^
AC haemorrhage	33.3	13	0.0881
Bleb leakage	0	0	>0.9999

AC, anterior chamber; BCVA; best-corrected visual acuity; NTG, normal-tension glaucoma; PMS, Preserflo MicroShunt. P-values were calculated using Fisher’s exact test. P-values <0.05 are bolded

Needling procedures were significantly less frequent in the NTG PMS group (*p* = 0.0013, log-rank test) and were required within the first 6, 12 and 24 months in 2% vs. 39%, 7% vs. 44%, and 17% vs. 48%, respectively. The number of needlings per patient-year follow-up was less than half in the NTG PMS group compared with the NTG XEN-45 group (0.096 vs. 0.261). After 2 years, incisional revisions were required in 2% of PMS and 0% of XEN-45 patients (*p* = 0.2562). Because only 2% incisional revisions occurred in the PMS group and none in the XEN-45 group, the Kaplan–Meier estimates for any bleb revision were nearly identical to those for needling alone (*p* = 0.0013). The numbers of incisional revisions and any bleb revision per patients year were 0.021 vs. 0 and 0.117 vs. 0.261 in the PMS and XEN-45 groups, respectively.

## Discussion

In this matched cohort study, PMS implantation achieved high rates of sustained low IOP control in patients with NTG. After 2 years, 69% of eyes achieved a ≥ 30% reduction in IOP with an IOP ≤ 12 mmHg without medication or incisional revision. Success rates were significantly higher compared with matched POAG PMS patients and consecutive NTG patients treated with the XEN-45 gel stent, requiring significantly fewer incisional bleb revisions and needlings, respectively. Notably, single-digit IOP (≤ 9 mmHg with ≥ 40% reduction) was achieved in 31% of NTG patients following PMS implantation, which was similar to the matched POAG cohort, but higher than the NTG XEN-45 group.

Our findings support previously reported 1-year outcomes of the PMS in NTG (*n* = 13) and extend these results by demonstrating sustained efficacy at 2 years, while 3-year results should be considered exploratory due to lower number of patients [13]. Pillunat et al. reported a 40% IOP reduction and 92.5% medication-free eyes one year after PMS implantation in NTG patients, which is comparable to the reductions observed in the present cohort (40% and 91%, respectively). Rates of AC reformation and AC haemorrhage were also similar between the studies (12% vs. 8% and 32% vs. 23%, respectively). However, approximately 23% of NTG patients in their cohort required needling within the first year compared with only 7% in our study. We also observed fewer bleb interventions, specifically incisional revisions, in NTG patients than in the matched POAG cohort. This contrasts with the findings of Pillunat et al., who did not report differences between NTG and unmatched POAG patients [13]. The same concentration and duration of MMC (0.2 mg/ml for 3 min) were used in both studies. The discrepancy may be explained by the larger cohort size in the current study. As bleb fibrosis is a major cause of failure in filtering surgery, the lower revision rates observed in NTG patients likely reflect the higher probability of maintaining low postoperative IOP. The same MMC concentration and application protocol and postoperative antifibrotic regimen and indication criteria for bleb revision were used for all PMS implantations regardless of diagnosis in the current study, reducing the risk of treatment-related bias in the PMS analysis.

Compared with NTG patients, the POAG cohort achieved a ≥ 30% IOP reduction with an IOP ≤ 12 mmHg in 45% of cases after 2 years, and a single-digit IOP ≤ 9 mmHg with a ≥ 40% reduction in 23%. The IOP reduction observed in our POAG cohort was 40% after 1 year, slightly higher than the 30% reported by Pillunat et al. [13]. Complete success rates in our POAG group for an IOP ≤ 15 mmHg with ≥ 25% reduction were 77% after 1 year and 64% after 2 years. These findings are comparable to or even higher than the approximately 54% success reported in a multicentre POAG study using a similar definition (IOP ≤ 15 mmHg with ≥ 20% reduction) [12]. Although POAG patients were matched to the NTG cohort for baseline characteristics, comparison to these outcomes support the representativeness of our POAG control group and the validity of comparison in the current study, and show that differences between diagnoses were not driven by lower success rates in the matched POAG group.

Glaucoma specialists may currently be less likely to treat NTG with the PMS, possibly due to the limited number of NTG cases reported in previous studies and the predominant use of the device in POAG [8, 9]. In POAG cohorts, median IOP in the range of 14–15 mmHg after one year have been reported [12]. Because lower IOP levels are typically required in NTG, this may discourage PMS use in these patients. Nevertheless, in the present study NTG patients achieved high surgical success even at lower IOP thresholds (≤ 12 mmHg) but not significantly different from the POAG control at very low thresholds (≤ 9 mmHg).

The robustness of the findings was confirmed through three sensitivity analyses. When incisional revisions were excluded from the failure definition, the association between NTG diagnosis and lower failure risk was preserved, with effect estimates of similar or greater magnitude for categories 2 and 3. This indicates that the observed differences were not driven by the higher revision rates in the POAG group. When absolute IOP thresholds were removed and success was defined by percentage IOP reduction alone, the association was similarly preserved and found for an IOP reduction ≥ 40%, demonstrating that NTG patients achieved greater proportional IOP reductions more frequently than POAG patients, independent of their lower baseline IOP. Even in the most conservative analysis combining both modifications, significant differences persisted for categories 1, 2 and 3. These consistent findings across multiple analytical approaches strengthen the conclusion that PMS implantation may achieve more favourable IOP outcomes in NTG compared with POAG and that the observed differences between cohorts were not solely driven by revision procedures. Importantly, the direction and magnitude of the effect estimates remained consistent across sensitivity analyses. Different outcomes between the two diagnoses could be driven by differences in tissue characteristics. NTG eyes have been shown to have significantly thinner conjunctival stroma compared with POAG eyes, which may reflect a lower fibrotic potential and contribute to more favourable bleb formation and lower revision rates [22]. Additionally, NTG eyes likely have less trabecular meshwork degeneration [23] and potentially preserved outflow facility through the trabecular meshwork, independent of the starting pressure.

The bleb revision rate after PMS implantation in our NTG cohort was 15%, which is comparable to the approximately 14% reported following trabeculectomy in NTG patients [3, 4]. Reported trabeculectomy success rates in NTG (defined as an IOP reduction of ≥ 30%) vary between 39.4% (qualified), 52% (complete and qualified), and 64.8% (complete) after 3 years [3, 4, 6]. In our PMS NTG cohort, complete and qualified success rates based on ≥ 30% IOP reduction were 72% and 75%, respectively, after 3 years (Fig. S3). When applying an additional IOP threshold (≤ 12 mmHg), success rates were 64% and 66%, which might be comparable to reported trabeculectomy outcomes.

Hypotony-related complications are a major concern following filtration surgery due to their potential impact on long-term visual acuity. In the present study, no long-term hypotony-associated complications were observed after PMS implantation in either NTG or POAG patients. This contrasts with reported rates of 23.6–30% following trabeculectomy in NTG patients [3, 5, 6]. Postoperative choroidal effusion occurred in 16% of NTG patients in our study and resolved within a median of 10 days after up to 2 AC injections of viscoelastic. Effusion rates were similar between NTG and POAG groups. We deliberately used a clinical definition of hypotony rather than a numerical threshold (e.g. <6 mmHg) because numerical definitions may underestimate true surgical success rates [17]. Rates of numerical hypotony beyond the initial 3-month period were negligible in both groups. No sight-threatening complications such as endophthalmitis, blebitis, or late-onset bleb leak were observed, whereas these complications have been reported in 0–8%, 5–6%, and 3–6% of NTG eyes after trabeculectomy [3–6].

In the second part of the study, PMS implantation was exploratorily compared with the XEN-45 Gel Stent in NTG patients. The PMS achieved significantly higher success rates for both low (≤ 12 mmHg) and very low (≤ 9 mmHg) postoperative IOP thresholds. As groups were unmatched, baseline imbalances were found for certain variables. Device type remained significantly associated with surgical failure in exploratory multivariable Cox models. Although follow-up was longer in the XEN group, sensitivity analyses using administrative truncation confirmed these findings.

Lower success rates in the XEN group were reflected by higher needling rates, with 39% of NTG eyes requiring needling within the first six months compared with only 2% after PMS implantation. A higher proportion of XEN-treated eyes also required medications during follow-up. The larger lumen of the PMS (70 μm vs. 45 μm) may allow greater aqueous outflow, achieving lower IOP thresholds and potentially reducing bleb fibrosis and improving long-term IOP control. Differences in bleb morphology between devices may further contribute to the differing efficacy and revision rates observed in this study [24]. Overall, these findings suggest higher efficacy of the PMS with lower revision burden compared with the XEN-45 in NTG patients. These differences are clinically relevant, as NTG patients often require low IOP levels that may not be reliably achieved with all MIBS procedures. Similar results have been reported in other comparative studies of other glaucoma subtypes that were not found in mixed glaucoma cohorts [25].

### Limitations

Several limitations should be acknowledged. First, the retrospective design introduces potential bias. However, it also reflects real-world practice, providing clinically relevant insights into the performance of PMS under routine conditions. Notably, most available studies on surgical outcomes in NTG are retrospective in nature, reflecting the challenges of conducting prospective trials in this limited population [26]. In the same line, although the sample size of the present study remains moderate, it compares favourably with the existing literature on surgical outcomes in NTG, where individual studies are typically limited to small cohorts. Most previous trabeculectomy studies in NTG have often included fewer than 50 eyes, and even recent meta-analyses are based on relatively small, pooled populations, frequently derived from predominantly Asian cohorts [26]. In this context, the present study provides one of the larger European cohorts evaluating surgical outcomes in NTG, thereby contributing additional data from a less frequently represented population [3, 5, 6]. Second, although matching was performed and PMS groups demonstrated excellent post-matching balance, residual bias from measured or unmeasured variables cannot be completely excluded, especially for the unmatched NTG analysis, which also had a lower number of patients in the XEN-45 group and should be considered exploratory. A direct comparison with a trabeculectomy cohort from our centre was not performed, as the number of NTG trabeculectomies during the study period was insufficient for a meaningful comparison. Finally, differences in follow-up duration between groups, particularly in the NTG comparison, may have influenced outcome assessment. Nevertheless, sensitivity analyses using administrative truncation yielded consistent results, supporting the robustness of the findings.

## Conclusion

In conclusion, this retrospective, single-surgeon, single-centre study found that PMS implantation achieved high rates of sustained low IOP in NTG patients with a favourable safety profile. Revision rates and hazard ratios for failure were significantly lower in NTG than matched POAG patients. In addition, PMS implantation showed higher success rates, lower needling rates, and lower failure risk when exploratorily compared with the XEN-45 Gel Stent in NTG patients. Long-term hypotony-related complications were not found in any group, although the sample size limits definitive safety conclusions. These findings suggest that the PMS may be a viable option for NTG patients, while mitigating the hypotony risk of traditional filtering surgery. Larger prospective studies are warranted to confirm these findings.

## Supplementary Information

Below is the link to the electronic supplementary material.

**Supplementary Material 1**: Supplementary Figures

**Supplementary Material 2**: Supplementary Tables

## Data Availability

The datasets analysed during the current study are available from the corresponding author upon reasonable request.
