# Supplementary material for: Achieving low intraocular pressure in normal-tension glaucoma with the Preserflo MicroShunt: comparison with primary open-angle glaucoma and the XEN-45 Gel Stent
Source: BMC Ophthalmol. 2026 Aug 21;26:518. doi: 10.1186/s12886-026-05254-1 (PMC13505109; doi:10.1186/s12886-026-05254-1)
Supplement: Supplementary file 1 [file 12886_2026_5254_MOESM1_ESM.docx]

**FIGURE S1.** Patient flow diagram.

MIBS, minimally invasive bleb surgery; NTG, normal-tension glaucoma; PMS, PRESERFLO MicroShunt; POAG, primary open-angle glaucoma; PSM, propensity score matching.

**FIGURE S2.** Kaplan–Meier survival curves for surgical success of the PMS in NTG versus POAG – sensitivity analysis 1: incisional bleb revisions not considered failure.

Same as Figure 2: Kaplan–Meier estimates were used to compare complete and qualified surgical success for category 1 (**A** and **D**), category 2 (**B** and **E**), and category 3 (**C** and **F**) between patients with NTG (orange) and POAG (blue) using the log-rank test. The 95% confidence intervals are represented in lighter orange and blue colours, respectively.

**FIGURE S3.** Kaplan–Meier survival curves for surgical success of the PMS in NTG versus POAG – sensitivity analysis 2: percentage IOP reduction only.

Same as Figure 2: Kaplan–Meier estimates were used to compare complete and qualified surgical success for category 1 (**A** and **D**), category 2 (**B** and **E**), and category 3 (**C** and **F**) between patients with NTG (orange) and POAG (blue) using the log-rank test. The 95% confidence intervals are represented in lighter orange and blue colours, respectively.

**FIGURE S4.** Kaplan–Meier survival curves for surgical success in of the PMS NTG versus POAG – sensitivity analysis 3: percentage IOP reduction only and incisional bleb revisions not considered failure.

Same as Figure 2: Kaplan–Meier estimates were used to compare complete and qualified surgical success for category 1 (**A** and **D**), category 2 (**B** and **E**), and category 3 (**C** and **F**) between patients with NTG (orange) and POAG (blue) using the log-rank test. The 95% confidence intervals are represented in lighter orange and blue colours, respectively.

**FIGURE S5.** Kaplan–Meier survival curves for surgical success in NTG following PMS versus XEN-45 implantation – sensitivity analysis: administrative right truncation at 24 months.

Same as Figure 4: Kaplan–Meier estimates were used to compare complete and qualified surgical success for category 1 (**A** and **D**), category 2 (**B** and **E**), and category 3 (**C** and **F**) between NTG eyes that received the PMS (orange) and XEN-45 implant (blue) using the log-rank test. The 95% confidence intervals are represented in lighter orange and blue colours, respectively.
