# Supplementary material for: Achieving low intraocular pressure in normal-tension glaucoma with the Preserflo MicroShunt: comparison with primary open-angle glaucoma and the XEN-45 Gel Stent
Source: BMC Ophthalmol. 2026 Aug 21;26:518. doi: 10.1186/s12886-026-05254-1 (PMC13505109; doi:10.1186/s12886-026-05254-1)
Supplement: Supplementary file 2 [file 12886_2026_5254_MOESM2_ESM.docx]

**Table S1.** Stratified univariate Cox regression – incisional revisions not considered failure

| **Success category** | **Events (n/N)** | **HR (95% CI)** | **P-Value (Cox)** |
| --- | --- | --- | --- |
| ≤9 mmHg, ≥40% (complete) | 68/100 | 0.66 (0.4-1.11) | 0.1169 |
| ≤9 mmHg, ≥40% (qualified) | 68/100 | 0.66 (0.4-1.11) | 0.1169 |
| ≤12 mmHg, ≥30% (complete) | 36/100 | **0.48 (0.23-0.99)** | **0.0476** |
| ≤12 mmHg, ≥30% (qualified) | 35/100 | **0.44 (0.21-0.91)** | **0.0277** |
| ≤15 mmHg, ≥25% (complete) | 19/100 | **0.29 (0.1-0.82)** | **0.0197** |
| ≤15 mmHg, ≥25% (qualified) | 16/100 | **0.28 (0.1-0.84)** | **0.0227** |

Same as Table 3, plus: In this sensitivity analysis, incisional bleb revisions were not considered surgical failure. Statistically significant results (p < 0.05) are shown in bold.

**Table S2.** Stratified univariate Cox regression – percentage IOP reduction only

| **Success category** | **Events (n/N)** | **HR (95% CI)** | **P-Value (Cox)** |
| --- | --- | --- | --- |
| ≥40% reduction (complete) | 45/100 | 0.58 (0.32-1.04) | 0.0696 |
| ≥40% reduction (qualified) | 44/100 | **0.53 (0.3-0.95)** | **0.0336** |
| ≥30% reduction (complete) | 30/100 | **0.45 (0.22-0.91)** | **0.0256** |
| ≥30% reduction (qualified) | 29/100 | **0.4 (0.2-0.81)** | **0.0112** |
| ≥25% reduction (complete) | 24/100 | **0.32 (0.13-0.76)** | **0.0104** |
| ≥25% reduction (qualified) | 21/100 | **0.32 (0.13-0.77)** | **0.0107** |

Same as Table 3, plus: In this sensitivity analysis, success was defined by percentage IOP reduction from baseline only, without absolute IOP thresholds. Statistically significant results (p < 0.05) are shown in bold.

**Table S3.** Stratified univariate Cox regression – percentage IOP reduction only, incisional revisions not considered failure

| **Success category** | **Events (n/N)** | **HR (95% CI)** | **P-Value (Cox)** |
| --- | --- | --- | --- |
| ≥40% reduction (complete) | 45/100 | 0.59 (0.32-1.06) | 0.0778 |
| ≥40% reduction (qualified) | 44/100 | **0.54 (0.3-0.97)** | **0.0377** |
| ≥30% reduction (complete) | 26/100 | **0.42 (0.19-0.91)** | **0.0285** |
| ≥30% reduction (qualified) | 25/100 | **0.37 (0.17-0.81)** | **0.0128** |
| ≥25% reduction (complete) | 19/100 | **0.28 (0.1-0.8)** | **0.0168** |
| ≥25% reduction (qualified) | 16/100 | **0.28 (0.1-0.82)** | **0.0205** |

Same as Table 3, plus: In this sensitivity analysis, success was defined by percentage IOP reduction from baseline only, without absolute IOP thresholds, and incisional bleb revisions were not considered surgical failure. Statistically significant results (p < 0.05) are shown in bold.

**Table S4.** Multivariable Cox regression for surgical failure following PMS versus XEN-45 implantation in NTG – administrative right truncation at 24 months

| **Variable** | **≤9 mmHg, ≥40% (complete)** | **≤9 mmHg, ≥40% (qualified)** | **≤12 mmHg, ≥30% (complete)** | **≤12 mmHg, ≥30% (qualified)** | **≤15 mmHg, ≥25% (complete)** | **≤15 mmHg, ≥25% (qualified)** |
| --- | --- | --- | --- | --- | --- | --- |
| PMS (reference: XEN-45) | **0.28 (0.15–0.51); <0.0001** | **0.28 (0.15–0.51); <0.0001** | **0.24 (0.11–0.51); <0.0001** | **0.25 (0.11–0.55); 0.001** | **0.26 (0.1–0.67); 0.006** | 0.46 (0.15–1.44); 0.183 |
| Number of preoperative medications | 1.02 (0.81–1.29); 0.852 | 1.02 (0.81–1.29); 0.852 | 0.87 (0.66–1.15); 0.337 | 0.88 (0.66–1.17); 0.375 | 0.99 (0.7–1.4); 0.957 | 1.09 (0.69–1.73); 0.706 |
| Pseudophakia | 1.27 (0.72–2.24); 0.411 | 1.27 (0.72–2.24); 0.411 | 0.79 (0.38–1.64); 0.528 | 0.56 (0.25–1.26); 0.159 | 1.05 (0.42–2.64); 0.922 | 0.99 (0.31–3.19); 0.991 |

Same as Table 5, plus: In this sensitivity analysis, administrative right truncation at 24 months was applied to account for the longer follow-up duration in the NTG XEN-45 group. Statistically significant results (p < 0.05) are shown in bold.
